# Surface Properties of a Novel Poly(vinyl alcohol) Film Prepared by Heterogeneous Saponification of Poly(vinyl acetate) Film

**DOI:** 10.3390/polym9100493

**Published:** 2017-10-09

**Authors:** Seong Baek Yang, Sung Hun Yoo, Joon Seok Lee, Jong Won Kim, Jeong Hyun Yeum

**Affiliations:** 1 Department of Bio-Fibers and Materials Science, Kyungpook National University, Daegu 41566, Korea; ysb@knu.ac.kr (S.B.Y.); enviro1234@naver.com (S.H.Y.); 2 Department of Textile Engineering & Technology, Yeungnam University, Gyeongsan 38541, Korea; leejs@ynu.ac.kr

**Keywords:** poly(vinyl alcohol) film, poly(vinyl acetate), solution casting, heterogeneous saponification, degree of saponification

## Abstract

Almost general poly(vinyl alcohol) (PVA) films were prepared by the processing of a PVA solution. For the first time, a novel poly(vinyl alcohol) (PVA) film was prepared by the saponification of a poly(vinyl acetate) (PVAc) film in a heterogenous medium. Under the same saponification conditions, the influence of saponification time on the degree of saponification (DS) was studied for the preparation of the saponified PVA film, and it was found that the DS varied with time. Optical microscopy was used to confirm the characteristics and surface morphology of the saponified PVA film, revealing unusual black globules in the film structure. The contact angle of the films was measured to study the surface properties, and the results showed that the saponified PVA film had a higher contact angle than the general PVA film. To confirm the transformation of the PVAc film to the PVA film, ^1^H nuclear magnetic resonance spectroscopy, X-ray diffraction measurements, differential scanning calorimetry, and Fourier-transform infrared spectroscopy were employed.

## 1. Introduction

Poly(vinyl alcohol) (PVA)—a polymer prepared by the saponification of poly(vinyl acetate) (PVAc)—has attracted considerable interest because of its many attractive properties, and is widely used in traditional industries, including food, pharmaceuticals, and biomedicine. Recently, PVA has gained increasing attention from researchers for biomedical applications such as eye drops, contact lenses, tissue adhesion barriers, and artificial cartilage, owing to its exclusive properties including biocompatibility, hydrophilicity, nontoxicity, and biodegradability [1]. It also has outstanding film-formation characteristics. Because of this property, combined with excellent chemical stability and hydrophilicity, it is blended with various synthetic and natural polymers like a water-soluble film and is used in food packaging [2]. Further, PVA films have various biomedical applications; for example, contact lenses, hemodialysis, artificial pancreases, synthetic vitreous humors, and cartilage and meniscus tissue replacement [3]. Several attempts have been made to develop a PVA-based film. PVA/graphene oxide nanocomposite film was prepared through organic/inorganic assemblies by Zhu et al. [4]. The preparation of metal-polymer hybrid nanocomposites from an aqueous solution of PVA and AgNO_3_ was reported by Gautam et al. [5]. Pérez-Juste et al. prepared PVA/gold-nanorod composite films by incorporating gold nanorods in PVA thin films and subsequently aligning the nanocomposite films by heating and stretching [6]. Silva et al. prepared a biodegradable and bioactive cashew-gum polysaccharide/PVA film and tested its potential for fungal inhibition [7]. Zhou et al. prepared and investigated the characteristics of a surface crosslinked thermoplastic starch/PVA blend film using ultraviolet irradiation [8].

Usually, the surface of the general PVA film is smooth if it has no inorganic material substituents and no surface treatment is performed. To modify the properties of PVA, several researchers have prepared PVA from PVAc microspheres using heterogeneous saponification. A simple and easy preparation of PVA/PVAc microspheres with a skin/core structure, involving suspension polymerization and heterogeneous surface saponification, was reported by Lee et al. [9]. They also developed a novel method for preparing syndiotactic PVA/poly(vinyl pivalate/vinyl acetate) microspheres using heterogeneous saponification [10]. Our group studied several PVA-based nanocomposite materials and investigated their characteristics and applications. We prepared PVAc/PVA/montmorillonite (MMT) nanocomposite microspheres by saponification of suspension-polymerized PVAc/MMT in a heterogeneous state [11] and investigated the effects of MMT on the polymerization and saponification rates of PVAc. We also prepared PVAc/MMT nanocomposite microspheres by suspension polymerization and converted it to PVA/MMT using heterogeneous saponification [12]. ^1^H-nuclear magnetic resonance (NMR) showed that fully hydrolyzed nanocomposite microspheres were successfully obtained. In our recent studies, we focused on the saponification of PVAc nanomaterials in a heterogeneous state. We developed a novel technique for preparing PVA/carbon nanotube nanomaterials by the saponification of synthesized PVAc/carbon nanotubes using suspension polymerization in a heterogeneous state. Further, we developed a technique for preparing PVA nanofibers by the saponification of electrospun PVAc nanofibers in a heterogeneous state [13]. In all of our aforementioned studies, we used a common method of heterogeneous saponification. There are no reports on the saponification of a PVAc film in a heterogeneous state to acquire PVA film, which was achieved for the first time by our group.

Our research group fabricated a PVA film by the saponification of a PVAc film, one of the most common precursors of PVA, in a heterogeneous state [10]. A solution-casting method was used to prepare the PVAc film. Unlike the general PVA films, our heterogeneously saponified film showed unusual black globules of different sizes in the film structure. In this work, we established a novel and simplistic technique to prepare PVA film from PVAc film, and a reasonable clarification was provided for the black globules appearing in the saponified film structure.

## 2. Experimental

Vinyl acetate (VAc) was procured from Sigma Aldrich and purified by washing with an aqueous solution of NaHSO_4_. The VAc monomer was mixed with 40% aqueous NaHSO_4_ solution to remove hydroquinone, which acts as a polymerization inhibitor, usually found in the VAc product. After washing, it was dried with CaCl_2_ (anhydrous) and then distilled in a low-pressure nitrogen atmosphere. 88% saponified PVA (Aldrich, *M*_n_ = 127,000 g/mol, St. Louis, MO, USA) was used as a suspension stabilizer. 2,2′-azobis(2,4-dimethylvaleronitrile) (ADMVN) was obtained from Wako Co (Tokyo, Japan) and used as an initiator after being twice recrystallized from methanol. For the heterogeneous saponification, we prepared an aqueous alkaline solution by mixing sodium hydroxide (NaOH, Duksan, Ansan, Korea), sodium sulfate (Na_2_SO_4_, Duksan), and methanol (MeOH, Duksan) in water. Deionized water was used for all the experiments.

For preparing the PVAc resin suspension, the polymerization of VAc was carried out in a chemical reactor. The 250 mL reactor was fitted with a condenser, and a dispersing agent was dissolved with constant stirring in a nitrogen atmosphere. After the completion of the degassing process, the VAc monomer and the ADMVN initiator were added at a polymerization temperature of 15 °C. The temperature was then increased to 60 °C. To isolate the globular PVAc particles, the reaction mixture was allowed to stand for a day after a prearranged reaction time. A PVAc film was prepared by the solution-casting technique. First, the PVAc solution was prepared by dissolving 10 wt % (*w*/*w*) PVAc in methanol under magnetic stirring for 2 h. The films were then prepared by casting the solution on glass slides, followed by the evaporation of methanol under a pressure of 40 cm Hg in a vacuum oven at room temperature for five days. The dry film was later stored in a plastic bag for the future experiments.

For the heterogeneous saponification of the PVAc film to the PVA film, a flask was fitted with a thermocouple, a reflux condenser, a dropping funnel, and a stirring device. For preparing the alkaline solution required for saponification, we used 10 g each of NaOH, Na_2_SO_4_, and MeOH, and 100 g of H_2_O. This alkaline solution condition is used for heterogeneous saponification of various microcapsules and nanofibers [10,11,12]. Under severe preparation conditions, the film turns brown and holes appear in the film. The saponification was continued and stopped at appropriate times, resulting in PVA forming on the surface of the PVAc film. After the requisite reaction time, the mixture was transferred into cold water and allowed to stand for 1 min, resulting in precipitation. The prepared saponified film was washed several times with water and then dried in vacuum at room temperature for 24 h. The thicknesses of the PVAc film, general PVA films, and saponified PVA film at different time intervals are shown in Table 1.

The film thickness was measured using a thickness gauge (IP65, Mitutoyo, Takatsu-ku, Japan). An optical microscope was used to examine the surface morphology of the saponified PVA films. A saponified PVAc film sample was dissolved in *d*_6_-dimethyl sulfoxide and proton NMR (^1^H NMR) spectroscopy (AVANCE III 500, Bruker, Germany) was carried out in the solution state. The degree of saponification (DS) of the saponified PVA film was determined by calculating the ratio of methyl and methylene proton peaks observed using ^1^H NMR spectroscopy. To measure the conversion of the PVAc film to the PVA film, an X-ray diffractometer (D/Max-2500, Rigaku, Tokyo, Japan) and a Fourier-transform infrared (FT-IR) spectrometer (Frontier, Perkin Elmer, Waltham, MA, USA) were used. The crystal-melting temperature of pure PVAc and the saponified PVA film were measured using a differential scanning calorimeter (DSC Q2000, TA instruments, New Castle, DE, USA). The contact angles of the films were measured using a contact-angle meter (DSA100, KRÜSS GmbH, Hamburg, Germany).

## 3. Results and Discussion

**Figure 1:**
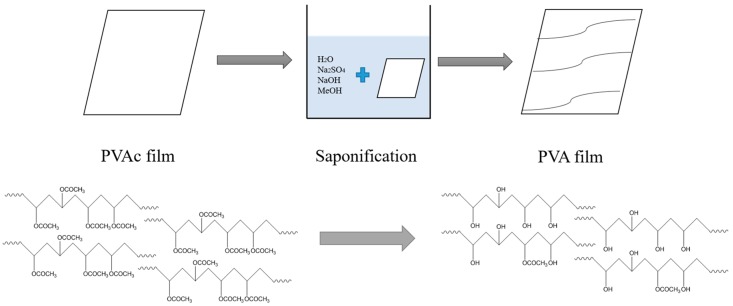
Schematic of the preparation of PVA by heterogeneous saponification.

Figure 1 shows a schematic of the method used for preparing the PVA film from the precursor PVAc film using heterogeneous saponification. Recently, we prepared the PVA nanofibers by the heterogeneous saponification of PVAc under different conditions and measured the ^1^H NMR spectra of the saponified PVA nanofibers. The results revealed that fully saponified PVA nanofibers were obtained with alkaline solutions containing 10 g each of NaOH, Na_2_SO_4_, and MeOH in 100 g of H_2_O [13]. We used a similar alkaline solution for the saponification of the PVAc film to prepare the PVA film, and the saponification was performed at 50 °C for 96 h.

**Figure 2:**
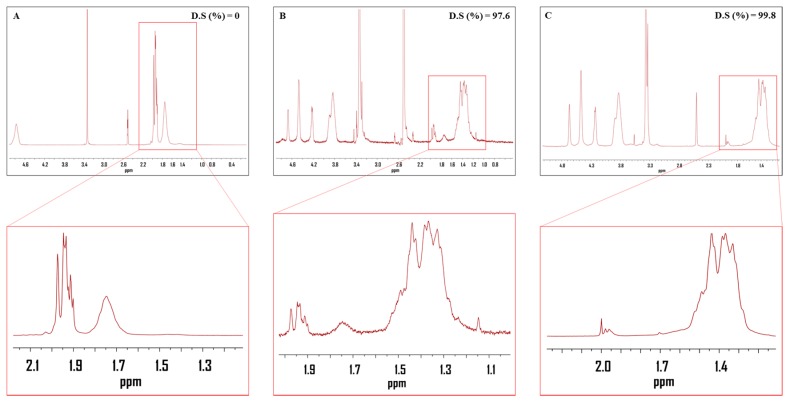
^1^H NMR spectra of the (**A**) PVAc film; (**B**) saponified PVAc film and (**C**) PVA film. The saponification time and temperature were 96 h and 50 °C, respectively. The saponification conditions were 10 g of NaOH, 10 g of Na_2_SO_4_, 10 g of MeOH, and 100 g of H_2_O, and the concentration of PVAc was 10 wt %.

The ^1^H NMR spectra of the pure PVAc film and fully saponified PVAc films (50 °C for 96 h) are shown in Figure 2. The DS of the PVA was calculated from the ratio of the methyl and methylene proton peaks (at 1.74 and 1.4 ppm) in the ^1^H NMR spectra. The nonexistence of methyl peaks indicated the complete conversion of the methyl group of PVAc to the methylene group of PVA (Figure 2B). A fully saponified PVA film was obtained under the saponification conditions of 10 g each of NaOH, Na_2_SO_4_, and MeOH in 100 g of H_2_O, at 50 °C for 96 h.

**Figure 3:**
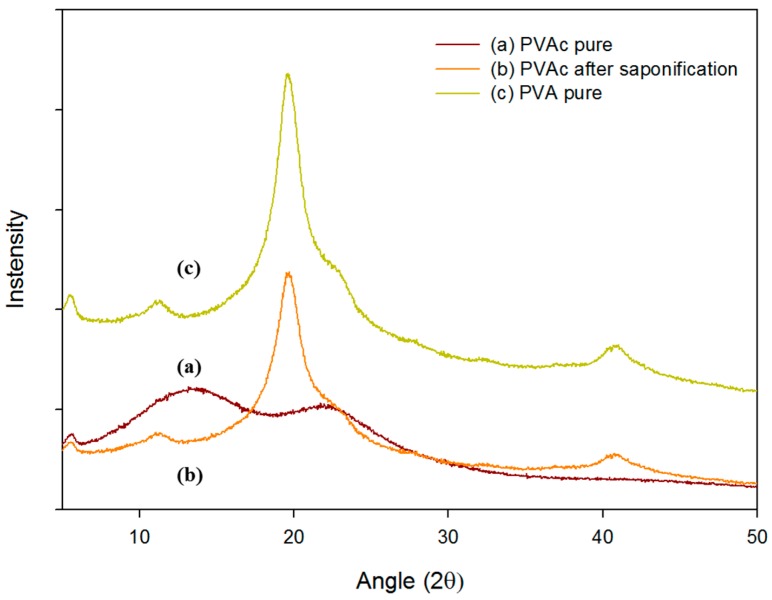
XRD patterns of the (**a**) pure PVAc film; (**b**) fully saponified PVAc film obtained by heterogeneous saponification at 50 °C for 96 h and (**c**) PVA pure film.

To observe the structural changes between the pure PVAc and fully saponified PVA film, X-ray diffraction (XRD) patterns were obtained. Figure 3 represents the diffractogram of the pure PVAc film and fully saponified PVA film prepared by heterogeneous saponification at 50 °C for 96 h. The two broad peaks at 13.5° and 22.5° clearly indicated the amorphous nature of the pure PVAc film. On the contrary, the saponified PVA film exhibited a sharp crystalline peak at 2θ = 19.8°. This observed peak is similar to the diffraction peak of the general PVA films. This proves the successful fabrication of the PVA film from the PVAc film. Pure PVA displays three characteristic diffraction peaks at 2θ = 13.5° (100 lattice plane), 19.8° (101 lattice plane), and 22.5° (200 lattice plane), as evident from the XRD results. These peaks correspond to the crystalline regions in the structure of the PVA [14,15].

**Figure 4:**
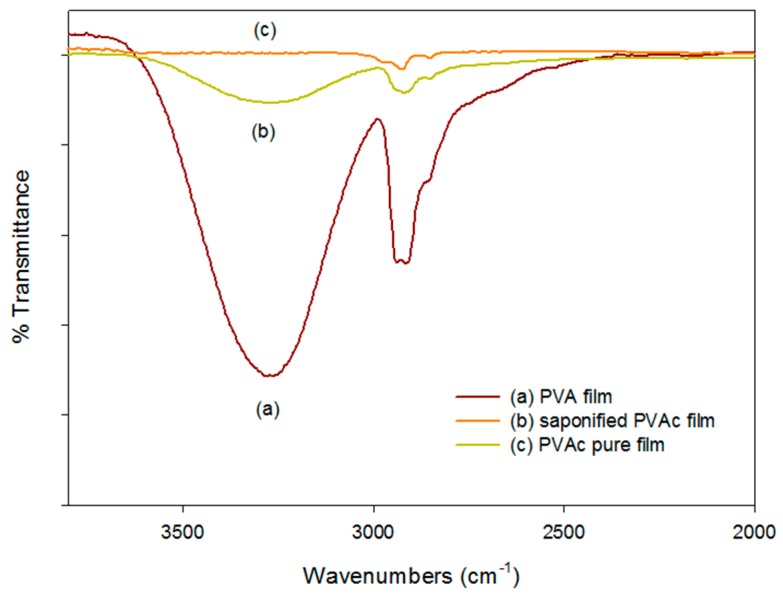
FT-IR spectra of the (**a**) general PVA film; (**b**) fully saponified PVAc film obtained by heterogeneous saponification at 50 °C for 96 h; and (**c**) pure PVAc film.

**Figure 5:**
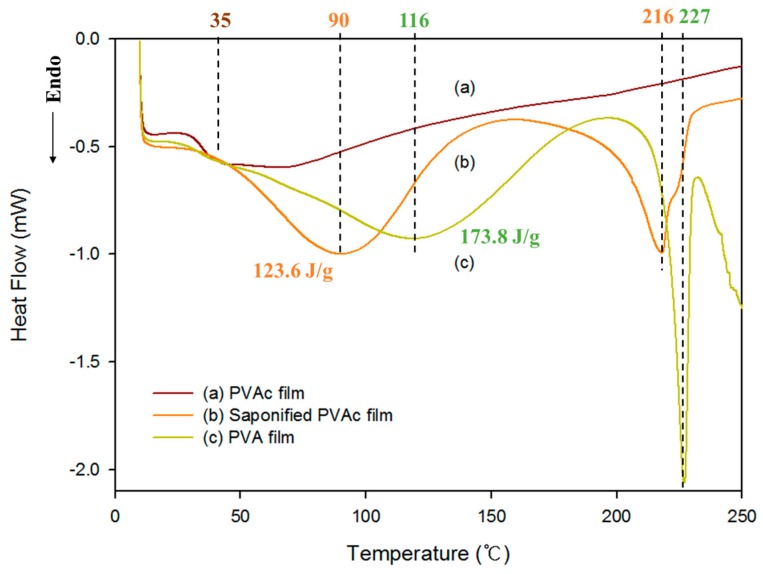
DSC curves for the (**a**) pure PVAc film; (**b**) PVAc film obtained by heterogeneous saponification at 50 °C for 96 h; and (**c**) general PVA film.

To characterize the effects of heterogeneous saponification on PVAc film, FT-IR analysis was performed. The FT-IR spectra of the general PVA film, the saponified PVAc film, and the pure PVAc film are shown in Figure 4. The spectra of the saponified PVA film showed a broad band in the range of 3000–3600 cm^−1^ due to the OH stretching vibration, which is similar to the absorption peak of general PVA, as described by different authors. This result provides further evidence of the saponification of the PVAc film. Differential scanning calorimetry (DSC) thermograms for the pure PVAc film, heterogeneously saponified PVAc film, and general PVA film are shown in Figure 5. An endothermic peak was observed at ~205 °C in the DSC curve for the heterogeneously saponified PVAc film at 50 °C for 96 h. Surprisingly, this peak is very similar to the peak obtained from general PVA film (~225 °C). This result further confirms the conversion of the PVAc film into the PVA film. The reason for the low melting temperature of the saponified PVAc film compared with the PVA film is that the backbone of the PVAc is partially damaged due to the strong alkaline environment of the saponification reaction. This could also happen when the OCOCH_3_ group is converted into the OH group in the already-filmed macroscopic structure, which results in poor crystallinity of the PVA.

**Figure 6:**
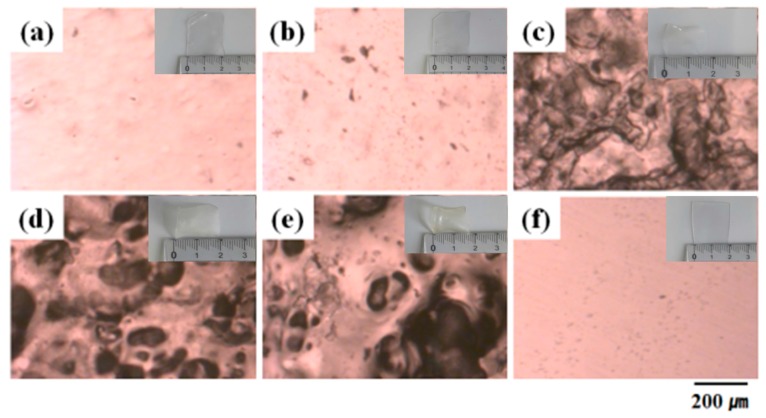
Optical microscopy images and macrographs of the (**a**) pure PVAc film; (**b**–**e**) PVAc films heterogeneously saponified at 50 °C; and (**f**) general PVA film. The saponification times and DS values were (**b**) 24 h and 0%; (**c**) 48 h and 39.4%; (**d**) 72 h and 91.6%; and (**e**) 96 h and 97.6%.

Our group prepared PVA microspheres or skin/core structures of PVAc microspheres by heterogeneous saponification [11,12]. However, the black globules were not observed in the saponified PVAc microspheres. Our group also established a novel and simplistic technique to prepare PVA nanofibers from PVAc nanofibers by heterogeneous saponification [13]. It was found that the surface roughness of the saponified PVA nanofiber increased. Even at the size of 1 micron, the chemical structure changed under the strong alkali environment, resulting in increased roughness. Therefore, the prepared saponified PVAc film is also expected to have black globules owing to the change in the chemical structure resulting from the strong alkali effect at the size of several hundred microns. Figure 6 shows that the size of the black globules increases with the progress of the saponification, and the average size of globules are (c) 32 μm, (d) 48 μm, and (e) 133 μm. More detailed morphologies were obtained by optical microscopy (Figure 6). Figure 6a shows the surface morphology of the pure PVAc film. The saponified PVAc film showed considerable change in the film morphology after 48 h of saponification (Figure 6c). These changes continued to happen even after 96 h. Changes in the film morphology were observed compared to the pristine PVAc film and general PVA film, where the DS was 97.6% and the presence of black globules of different sizes on the white surface of the film was observed (Figure 6e). The mass of the film remained constant after saponification but the volume decreased. Therefore, the film density after saponification increased relative to the film density before saponification. The possible reasons for the unusual product shape are (i) the increase in film density after saponification; and (ii) PVA skins dissolution in the aqueous saponification solution. Even if the number of chains remain constant, the density of the polymer chain becomes different if the chain ends are clustered or the chain ends are small. The PVAc molecule is a chain of PVA molecules that can react with strong alkali. When the reaction proceeds violently in the alkali environment, the size of the chain ends is small, or the side chains of the polymer are less distributed, pitting of the chain is also possible. This enhances the reaction rate as the DS increases.

**Figure 7:**
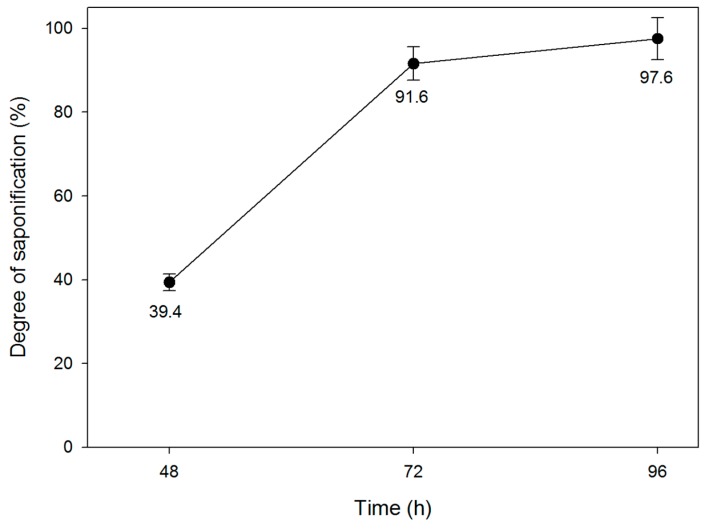
Effect of the saponification time on the DS of the PVAc film. (The saponification conditions were 10 g of NaOH, 10 g of Na_2_SO_4_, 10 g of MeOH, and 100 g of H_2_O at 50 °C).

Time plays an important role in obtaining fully saponified PVA. The effect of the saponification time on the DS of the PVAc film is illustrated in Figure 7. The values of the DS increased remarkably with the saponification time and reached 97.6% after 96 h of saponification. The saponification conditions were 10 g each of NaOH, Na_2_SO_4_, and MeOH in 100 g of H_2_O at 50 °C.

The surface properties of the films were investigated by contact-angle measurements. Table 2 shows the results obtained for different DS values of the films. The saponified PVA film with a higher DS showed a decrease in the contact angle compared with the pure PVAc film. The decrease in contact angle of the alkali-treated PVA films due to erosion and the pits probably changed the roughness after the saponification. On the other hand, the saponified PVA film had a higher contact angle compared to the general PVA film. This shows that the contact angle of saponified PVAc is affected by the roughness of the film surface rather than the hydrophilicity of the saponified PVAc, and that proves that the surface roughness of the saponified PVAc film has changed.

## 4. Conclusions

Generally, PVA film is prepared by solution casting using PVA solution. However, in this work, a fully saponified PVA film was successfully fabricated by the heterogeneous saponification of a PVAc film. An optical microscopic study revealed that black globules of different sizes were present in the film structure. The surface properties of the films were investigated by contact-angle measurement, and the results showed that the saponified PVA film with a higher DS showed a lower contact angle than the pure PVAc film. The formation of a fully saponified PVA film was confirmed by ^1^H-NMR studies. The experimental results indicated that the DS depends on the reaction time for the same saponification conditions, and the detailed structure of the PVA film was established using XRD studies. FT-IR and DSC measurements provided further evidence of the conversion of the PVAc film into the PVA film.

## Figures and Tables

**Table 1:** Thicknesses of the PVAc film, general PVA film, and saponified PVA film at different time intervals.

Film sample	Time of saponification (h)	Thickness (μm)
Pure PVAc	-	175 ± 12
Saponified PVA	24	174 ± 14
Saponified PVA	48	172 ± 12
Saponified PVA	72	164 ± 13
Saponified PVA	96	168 ± 15
General PVA	-	177 ± 12

**Table 2:** Contact angles of the PVAc film, general PVA film, and saponified PVA film with respect to the DS.

Film sample	Average DS (%)	Contact angle (°)
Pure PVAc	0	65.5 ± 2.4
Saponified PVA	39.4	41.3 ± 1.6
Saponified PVA	97.6	20.7 ± 2.1
General PVA	99.9	6.3 ± 0.4
